# Activin A inhibits vascular endothelial cell growth and suppresses tumour angiogenesis in gastric cancer

**DOI:** 10.1038/bjc.2011.348

**Published:** 2011-09-06

**Authors:** H Kaneda, T Arao, K Matsumoto, M A De Velasco, D Tamura, K Aomatsu, K Kudo, K Sakai, T Nagai, Y Fujita, K Tanaka, K Yanagihara, Y Yamada, I Okamoto, K Nakagawa, K Nishio

**Affiliations:** 1 Department of Genome Biology, Kinki University School of Medicine, 377-2 Ohno-higashi, Osaka-Sayama, Osaka 589-8511, Japan; 2 Department of Medical Oncology, Kinki University School of Medicine, 377-2 Ohno-higashi, Osaka-Sayama, Osaka 589-8511, Japan; 3 Laboratory of Health Sciences, Department of Life Sciences, Yasuda Women's University Faculty of Pharmacy, 6-13-1 Yasuhigashi, Asaminami-ku, Hiroshima 731-0153, Japan; 4 Department of Medical Oncology, National Cancer Center Hospital, Tsukiji 5-1-1, Chuo-ku, Tokyo 104-0045, Japan

**Keywords:** activin A, p21^CIP1/WAF1^, angiogenesis, gastric cancer

## Abstract

**Background:**

Activin A is a multi-functional cytokine belonging to the transforming growth factor-*β* (TGF-*β*) superfamily; however, the effect of activin A on angiogenesis remains largely unclear. We found that *inhibin β A subunit* (*INHBA*) mRNA is overexpressed in gastric cancer (GC) specimens and investigated the effect of activin A, a homodimer of INHBA, on angiogenesis in GC.

**Methods:**

Anti-angiogenic effects of activin A via p21 induction were evaluated using human umbilical vein endothelial cells (HUVECs) *in vitro* and a stable *INHBA*-introduced GC cell line *in vivo*.

**Results:**

Compared with TGF-*β*, activin A potently inhibited the cellular proliferation and tube formation of HUVECs with induction of p21. A promoter assay and a chromatin immunoprecipitation assay revealed that activin A directly regulates p21 transcriptional activity through Smads. Stable p21-knockdown significantly enhanced the cellular proliferation of HUVECs. Notably, stable p21-knockdown exhibited a resistance to activin-mediated growth inhibition in HUVECs, indicating that p21 induction has a key role on activin A-mediated growth inhibition in vascular endothelial cells. Finally, a stable *INHBA*-introduced GC cell line exhibited a decrease in tumour growth and angiogenesis *in vivo*.

**Conclusion:**

Our findings highlight the suppressive role of activin A, unlike TGF-*β*, on tumour growth and angiogenesis in GC.

Activins are homodimers formed by the assembly of two closely related inhibin *β* subunits, *β*A and *β*B, which generate three isoforms, activin A (*β*A–*β*A), activin B (*β*B–*β*B), and activin AB (*β*A–*β*B) (Chen *et al*, 2002). Activin A is a member of the transforming growth factor (TGF-*β*) superfamily and shares the Smad intracellular signalling proteins with TGF-*β* (Shi and Massague, 2003). Activin A binds to activin type II receptors, ActR-II and ActR-IIB, and the ligand/type II receptor complex then recruits, binds, and phosphorylates the type I receptor ActR-IB, also known as activin receptor-like kinase 4 (ALK4), resulting in the propagation of the signal downstream. The activation of ALK4 kinase phosphorylates and activates the cytoplasmic signalling molecules Smad2 or Smad3, and a specific activated Smad complex then translocates and accumulates in the nucleus, where it is involved in the transcriptional regulation of target genes (Shi and Massague, 2003).

Activins have been found to control a wide spectrum of biological effects, such as cellular growth and developmental differentiation in many cell types, although it was originally described as a regulator of follicle-stimulating hormone release from the anterior pituitary (Ying, 1988; Dawid *et al*, 1992; Munz *et al*, 2001; Shav-Tal and Zipori, 2002). Recently, activin A has been reported as an essential growth factor involved in embryonic stem cell renewal and pluripotency (Xiao *et al*, 2006; Jiang *et al*, 2007). In general, activin A causes growth inhibition in epithelial cells ranging from many normal mesenchymal and haematopoietic cells to a variety of cancer cells. In addition, activin A not only inhibits cell proliferation, but it also induces apoptosis in multiple cells and tissues. For example, activin A inhibits the cellular proliferation of breast cancer T47D cells by enhancing the expression of p15 cyclin-dependent kinase (cdk) inhibitors, and the overexpression of activin A in human prostate cancer LNCaP cells inhibited proliferation, induced apoptosis, and decreased the tumourigenicity of these cells (Zhang *et al*, 1997; Burdette *et al*, 2005). In addition, activin A also reportedly exerts a tumour suppressor function in human neuroblastoma cells (Panopoulou *et al*, 2005). In angiogenetic roles, emerging evidence has demonstrated that TGF-*β*, the other superfamily member, may definitely stimulate angiogenesis during the late stage of cancer (Jakowlew, 2006), while neuroblastoma cells with restored activin A expression exhibit a decreased tumour growth and reduced vascularity (Panopoulou *et al*, 2005). Collectively, whether activin A inhibits angiogenesis in other cancers in addition to its underlying mechanism resulting in the growth inhibition of vascular endothelial cells remains unclear.

We previously performed a microarray analysis of paired gastric cancer (GC) and non-cancerous gastric mucosa samples and identified the overexpression of *INHBA* in the GC samples (Yamada *et al*, 2008 and unpublished data). Based on this finding of *INHBA* overexpression and accumulating evidence of the role of TGF-*β* in angiogenesis, we focused on the role of activin A in angiogenesis in GC in the present study.

## Materials and methods

### Antibodies and ligands

The following antibodies were used: anti-p21, anti-cdk2, anti-cyclin D, anti-phospho-Rb, anti-Smad2, anti-phospho-Smad2, anti-Smad3, anti-Smad4, and secondary antibodies (Cell Signaling, Beverly, MA, USA); anti-*β*-actin (Santa Cruz Biotechnology, Santa Cruz, CA, USA); and a mouse anti-CD31 monoclonal antibody (BD Biosciences, San Jose, CA, USA). Recombinant human activin A and TGF-*β*1 were purchased from R&D Systems (Minneapolis, MN, USA). The Alk4/Alk5/Alk7-specific inhibitor SB341542 was purchased from Sigma (St Louis, MO, USA).

### Cell lines and cultures

58As1, 44As3, Okajima, KATOIII, TU-KATOIII, MKN1, MKN7, MKN28, and MKN74 cell lines were cultured in RPMI-1640 medium (Sigma) with 10% heat-inactivated fetal bovine serum (FBS; Gibco BRL). The HEK293 (Human Embryonic Kidney cell line 293) cell lines were cultured in DMEM medium (Sigma) with 10% heat-inactivated FBS. Human umbilical vein endothelial cells (HUVECs) were purchased from Kurabo (Osaka, Japan) and maintained in Humedia (Kurabo) supplemented with 2% FBS, 5 ng ml^–1^ FGF-2, 10 ng ml^–1^ EGF, 10 *μ*g ml^–1^ heparin, 1 *μ*g ml^–1^ hydrocortisone, and antibiotics. The cell lines were maintained in a 5% CO_2_-humidified atmosphere at 37°C.

### Patients and samples

The methods were described previously (Yamada *et al*, 2008). This study was approved by the institutional review board, and written informed consent was obtained from all the patients.

### Plasmid construction, viral production, and stable transfectants

The methods used in this section have been previously described (Kaneda *et al*, 2010). Briefly, the cDNA fragment encoding human full-length INHBA was isolated using PCR and Prime STAR HS DNA polymerase (TaKaRa, Otsu, Japan) with 5′-GGG AAT TCG CCA GGA TGC CCT TGC TTT GGC TGA GAC-3′ and 5′-GCC CTC GAG GGC AAC TCT ATG AGC ACC CAC ACT CC-3′ sense and antisense primers, respectively. Stable transfectants expressing EGFP or INHBA in TU-KATOIII cells were designated as TK3/EGFP and TK3/INHBA. Short hairpin RNA (shRNA)-targeting p21 was constructed using oligonucleotides encoding small interfering RNA directed against p21 and a non-specific target as follows: 5′-CTA AGA GTG CTG GGC ATT TTT-3′ for p21 shRNA and 5′-TGT TCG CAG TAC GGT AAT GTT-3′ for control shRNA. They were then cloned into an RNAi-Ready pSIREN-RetroQZsGreen vector (Clontech, Mountain View, CA, USA) according to the manufacturer's protocol. The stable transfectants expressing shRNA-p21 or shRNA-scramble in HUVECs were designated as HUVEC/sh-p21 and HUVEC/sh-Scr, respectively.

### Real-time reverse transcription PCR and western blot analysis

The methods used in this section have been previously described (Matsumoto *et al*, 2009). The primers used for real-time RT–PCR were as follows: *INHBA* forward, 5′-CAT TGC TCC CTC TGG CTA TCA T-3′ and reverse, 5′-GCA CAC AGC ACG ATT TGA GGT T-3′ GAPD forward, 5′-GCA CCG TCA AGG CTG AGA AC-3′ and reverse, 5′-ATG GTG GTG AAG ACG CCA GT-3′. The densitometry data from the western blot analysis were quantified automatically using Multigauge Ver. 3.0 (Fujifilm, Tokyo Japan). The densitometry data were normalised by *β*-actin and is shown above the western blot as a ratio of that in the control sample.

### ELISA

A total of 10^6^ cells from each of the GC cell lines TK3/EGFP and TK3/INHBA were cultured in normal medium for 12 h and the medium was replaced with a serum-free medium. After 12 h of culture, the medium was collected, centrifuged to remove floating cells, and used for analysis. The concentration of activin A described above was determined using a human activin A DuoSet ELISA Development kit (R&D Systems), according to the manufacture's instructions.

### Cell proliferation assay

HUVECs were plated at a density of 3 × 10^3^ cells in 96 wells in growth medium overnight. The cells were then stimulated with the vehicle, activin A, or TGF-*β* at the indicated concentrations for 72 h (Figures 2A and 5C) or the indicated time (Figure 5B). The experiment was performed using an MTT assay in triplicate. The methods have been previously described (Kaneda *et al*, 2010).

### Tube formation assay

A 96-well plate was coated with Matrigel (BD Biosciences) avoiding bubble formation and was incubated at 37°C for 30 min to allow the Matrigel to solidify. HUVECs (2 × 10^4^ cells per well) were pretreated with 10 ng ml^–1^ of activin A or 1 ng ml^–1^ of TGF-*β* for 48 h and then were plated onto the Matrigel-coated plate. After 16 h of incubation, the HUVECs were photographed using fluorescence microscopy (IX71; Olympus, Tokyo, Japan).

### Luciferase reporter assay

The human p21 promoter-containing reporter vector was constructed according to a previously described method (Kaneda *et al*, 2010). Briefly, a 2.4-kb section of the p21 promoter region was subcloned into a luciferase reporter vector, pGL4.14 (Promega, Madison, WI, USA). All the sequences were verified using DNA sequencing. The empty and p21 promoter-containing reporter vectors were designated as pGL4.14-mock and pGL4.14-p21, respectively. The results were normalised to co-transfected *β*-galactosidase activity and are representative of at least three independent experiments.

### Chromatin immunoprecipitation

Chromatin immunoprecipitation (ChIP) was performed using the SimpleChIP Enzymatic Chromatin IP Kit (Cell Signaling Technology) according to the manufacturer's protocol. The Smad-binding region (SBR) of the p21 promoter was amplified using the following primers: forward 5′-TTC ATT GTG AAG CTC AGT ACC AC-3′ and reverse 5′-TCA AAT GTC CAG CAG AGG ACA G-3′. As a negative control, the GAPDH second intron promoter was amplified using the following primers: (forward) 5′-AAT GAA TGG GCA GCC GTT AG-3′ and (reverse) 5′-AGC TAG CCT CGC TCC ACCTGA C-3′.

### Xenograft studies and immunohistochemical staining

Nude mice (*BALB/c nu/nu*; 6-week-old females; CLEA Japan Inc., Tokyo, Japan) were used for the *in vivo* studies and were cared for in accordance with the recommendations for the Handling of Laboratory Animals for Biomedical Research, as compiled by the Committee on Safety and Ethical Handling Regulations for Laboratory Animals Experiments, Kinki University. The ethical procedures followed and met the requirements of the UKCCCR guidelines. To evaluate tumour growth and tumour angiogenesis, the cell suspensions of 1 × 10^7^ TK3/EGFP or TK3/INHBA cells in 0.1 ml PBS were subcutaneously injected into the left or right flanks of nude mice (*n*=5), respectively. The tumour volume was calculated as the length × width^2^ × 0.5. The tumour volume was assessed every week. At the end of the experiment, the mice were killed and the xenografts were resected, fixed in 10% buffered formalin for 10 h, and processed for histological analysis. The immunohistochemical staining methods have been previously described (Kaneda *et al*, 2010).

### Statistical analysis

The statistical analyses were performed using Microsoft Excel (Microsoft, Redmond, WA, USA) to calculate the average, s.d., and results of a Student's *t*-test. A *P*-value <0.05 was considered statistically significant.

## Results

### *INHBA* mRNA is overexpressed in GC

**Figure 1:**
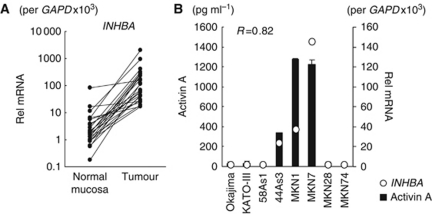
Overexpression of *INHBA* (inhibin *β* A) mRNA in GC specimens and secretion of its homodimer form, activin A, in GC cell lines. (**A**) The mRNA expressions of *INHBA* in 24 GC and paired non-cancerous gastric mucosa samples were determined using real-time RT–PCR. (**B**) A strong correlation between the expressions of *INHBA* mRNA and activin A protein was observed in GC cell lines, as determined using real-time RT–PCR and an ELISA, respectively. Rel mRNA: normalised mRNA expression levels (*INHBA/GAPD* × 10^3^). The correlation coefficient is shown in the figure.

A real-time RT–PCR analysis revealed that *INHBA* mRNA was overexpressed an average of 37-fold higher in 24 GC specimens than in paired non-cancerous mucosa samples (*P*=0.014; Figure 1A). The average levels of *INHBA* mRNA expression in the GC and paired non-cancerous mucosa samples were 236±422 and 6.0±16.0 ( × 10^3^/*GAPD*), respectively. Since INHBA/activins proteins are multi-functional ligands and its superfamily member, TGF-*β*, is closely involved in angiogenesis, we speculated that the overexpression of *INHBA* may have some role in tumour biology. Thus, we focused on the effect of activin A on tumour angiogenesis.

*INHBA* overexpression likely leads to the overexpression of its homodimer form, activin A; therefore, we evaluated the correlation between mRNA expression and secreted activin A protein expression in nine GC cell lines. The expressions of both *INHBA* mRNA and activin A protein were increased in 44As3, MKN1, and MKN7 cells but were very low in the other cell lines (Figure 1B). These mRNA and protein expressions were strongly correlated (*R*=0.82), indicating that *INHBA* overexpression in GC leads to the overexpression of activin A.

### Activin A potently inhibits cellular proliferation in vascular endothelial cells

**Figure 2:**
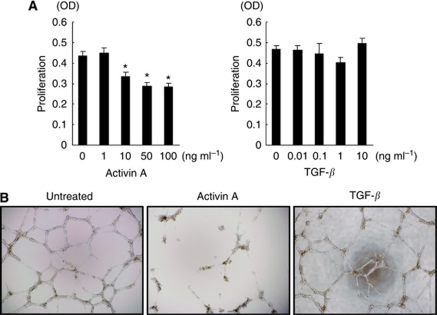
Activin A potently inhibits the proliferation and tube formation of HUVECs. (**A**) HUVECs were cultured in 96-well plates and stimulated with the indicated doses of activin A or TGF-*β* for 72 h. The cell proliferation was assayed using an MTT assay. Columns: mean of independent triplicate experiments. Bars: s.d. ^*^P<0.05. (**B**) Effect of activin A on tube formation in HUVECs. HUVECs were cultured with normal medium (untreated) or activin A (10 ng ml^–1^) or TGF-*β* (1 ng ml^–1^) containing medium for 48 h and the cells were seeded in 96-well culture plates (1.5 × 10^4^ cells per well) precoated with 80 *μ*l Matrigel and cultured with normal medium (untreated) or activin A (10 ng ml^–1^) or TGF-*β* (1 ng ml^–1^). After 16 h of incubation, the wells were photographed using a microscope.

We examined the effect of activin A, compared with TGF-*β*, on cellular proliferation using HUVECs. TGF-*β* slightly decreased cellular proliferation at a dose of 1 ng ml^–1^, while a higher dose of TGF-*β* (10 ng ml^–1^) tended to increase proliferation (Figure 2A). In contrast, activin A potently and dose-dependently decreased cellular proliferation at a dose of 10–100 ng ml^–1^ (Figure 2A). In addition, a tube formation assay showed that activin A, but not TGF-*β*, inhibited tube formation in HUVECs (Figure 2B). These results indicate that activin A and TGF-*β* have quite different effects on the cellular proliferation of vascular endothelial cells.

### Activin A mediates the persistent phosphorylation of Smad2 and p21 induction in HUVECs

**Figure 3:**
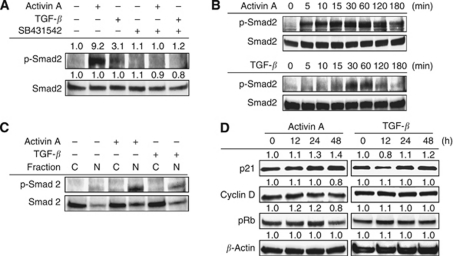
Activin A mediates the persistent phosphorylation of Smad2 and p21 induction in HUVECs. (**A**) HUVECs were treated with or without 2 *μ*M of SB341542 for 30 min, then stimulated with activin A (10 ng ml^–1^) or TGF-*β* (1 ng ml^–1^) for 1 h. The phosphorylation and expression levels of Smad2 were evaluated using western blot. (**B**) Time-course analysis with activin A or TGF-*β*-induced Smad2 phosphorylation. HUVECs were stimulated with 10 ng ml^–1^ activin A or 1 ng ml^–1^ TGF-*β* for the indicated time periods. (**C**) Activin A or TGF-*β*-induced nuclear translocation of Smad2. HUVECs were stimulated with or without activin A (10 ng ml^–1^) or TGF-*β* (1 ng ml^–1^) for 1 h. Nuclear and cytosolic protein fractions were then analysed using a western blot analysis. C= cytosolic fraction; N=nuclear fraction. (**D**) Expression changes of cell cycle-related proteins by stimulation with activin A (10 ng ml^–1^) or TGF-*β* (1 ng ml^–1^) for the indicated time period in HUVECs. A western blot analysis was performed using anti-p21, cyclin D, and phosphorylated Rb antibodies. *β*-Actin was used as an internal control.

Activin A and TGF-*β* inhibited the cellular growth of HUVECs in different manners; therefore, we examined the downstream signalling under activin A or TGF-*β* stimulation. Based on the many previous studies and our data for cellular growth inhibition (Figure 2A), we used 10 ng ml^–1^ of activin A and 1 ng ml^–1^ of TGF-*β* as moderate doses to compare the effects of activin A and TGF-*β*. Activin A (10 ng ml^–1^) strongly increased the phosphorylation levels of Smad2, compared with TGF-*β* (1 ng ml^–1^), and the effects were cancelled by the Alk4/Alk5/Alk7 inhibitor SB341542 (Figure 3A). Interestingly, activin A persistently increased the phosphorylation of Smad2 from 5 min to over 3 h, while TGF-*β* increased the phosphorylation during a shorter period of from 15 to 60 min (Figure 3B). In addition, activin A strongly induced the nuclear translocation of phosphorylated-Smad2 and Smad2, while TGF-*β* induced a milder effect (Figure 3C). These results suggest that activin A activates Smad signalling more potently than TGF-*β* in HUVECs.

Next, we evaluated the expression levels of cell cycle-related proteins to investigate the difference in the growth inhibitory effects. A western blot analysis revealed that the expression of cyclin D1 and the phosphorylation levels of Rb were decreased by activin A stimulation after 48 h of stimulation, while TGF-*β* showed a weak effect consistent with the results for growth inhibition (Figure 3D).

p21^CIP1/WAF1^ is a major cdk inhibitor and the hallmark of the cytostatic role of TGF-*β* (Weiss, 2003). TGF-*β* is known to increase p21 expression (Datto *et al*, 1995; Hu *et al*, 1998; Pardali *et al*, 2000), but the regulation of p21 by activin A remains largely unclear, especially in vascular endothelial cells. We found that p21 expression was increased by both activin A and TGF-*β* stimulation at 12–48 h in HUVECs (Figure 3D); therefore, we speculated that p21 may have a role in activin A-mediated growth inhibition and cell-cycle progression in vascular endothelial cells.

### Activin A directly regulates p21 transcriptional activity through Smads

**Figure 4:**
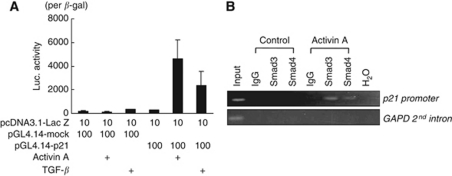
Activin A directly increases *p21* promoter activity. (**A**) *p21* promoter activity was determined using a luciferase assay. HEK293 cells were transiently transfected with luciferase vectors containing empty or p21 promoters (pGL4.14-mock or pGL4.14-p21) and then stimulated with activin A (10 ng ml^–1^) or TGF-*β* (1 ng ml^–1^) for 24 h. The data were normalised by *β*-galactosidase activity of co-transfected with the Lac Z vector in at least three independent experiments. Columns: mean of experiments. Bars: s.d. (**B**) ChIP of activin A-induced Smads on the promoter of *p21*. HUVECs were treated with 10 ng ml^–1^ of activin A for 1 h and collected for analysis. The data show the PCR amplification of the *p21* promoter using inputs (1% of chromatin used for ChIP) or ChIPs using smad3 or smad4 antibodies as templates. Primers to the *GAPDH* promoter were used as a negative control. IgG: non-specific IgG as a control.

A recent report demonstrated that TGF-*β* increases the binding of Smad2/3 and Smad4 to a distal portion of the p21 promoter, in which a SBR contains four Smad-binding elements (Seoane *et al*, 2004). To determine whether activin A regulates p21 expression at the transcriptional level, we performed a luciferase reporter assay. Activin A stimulation markedly increased the p21 promoter activity to 16.9-fold, compared with a control (Figure 4A). TGF-*β* also increased the promoter activity to 8.6-fold, but the effect was about half of that of activin A. A ChIP assay showed that activin A increased the binding of Smad2/3 and Smad4 to the SBR on the p21 promoter (Figure 4B). These results indicate that activin A directly regulates the p21 transcriptional activity through Smads.

### p21 induction has a key role on activin A-mediated growth inhibition in vascular endothelial cells

**Figure 5:**
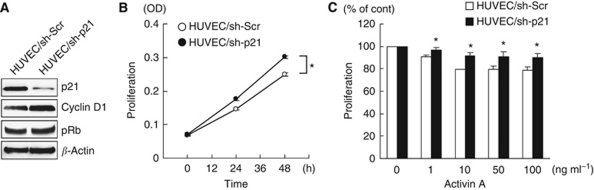
p21 induction has a key role in activin A-mediated growth inhibition in vascular endothelial cells. (**A**) Stable HUVECs transfected with control or p21-knockdown viral shRNA vector (HUVEC/sh-Scr and HUVEC/sh-p21) were evaluated using a western blot analysis using p21, cyclin D, and phospho-Rb antibodies. *β*-Actin was used as an internal control. (**B**) The cell growth curves of HUVEC/sh-Scr and HUVEC/sh-p21 cells were evaluated using an MTT assay in triplicate experiments. ^*^P<0.05. (**C**) Growth inhibition of HUVEC/sh-Scr and HUVEC/sh-p21 cells by activin A stimulation. The cells were stimulated with the indicated doses of activin A for 48 h. Cell growth was determined using an MTT assay. Data were shown as the percentage of activin A-untreated controls and are shown as the mean±s.d. of at least three independent experiments. ^*^P<0.05.

To evaluate the role of p21 induction on activin A-mediated growth inhibition, we examined the cellular growth of stable p21-knockdown (HUVEC/sh-p21) or control (HUVEC/sh-Scr) HUVECs using viral shRNA-targeting p21 or shRNA-scramble vectors (Kaneda *et al*, 2010). Interestingly, the knockdown of p21 significantly increased the cellular proliferation, compared with a control, in HUVECs, indicating that p21 has a growth inhibitory role in HUVECs (Figure 5B). Furthermore, stable p21-knockdown HUVECs were significantly resistant to activin A-mediated growth inhibition, compared with a control, although not completely resistant (Figure 5C). Our new findings demonstrate that p21 induction at least partially has a key role in activin A-mediated growth inhibition in vascular endothelial cells.

### Effect of activin A activity on cellular proliferation in GC cell lines

**Figure 6:**
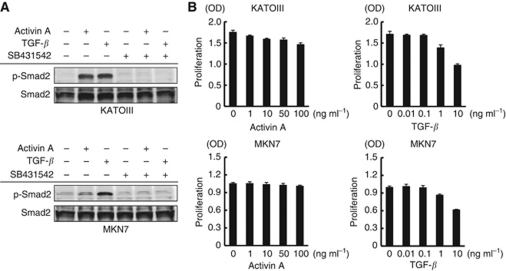
Activin A inhibits the cellular proliferation of KATOIII cells but does not inhibit the proliferation of MKN7 cells. (**A**) GC cell lines KATOIII and MKN7 were treated with or without 2 *μ*M of SB341542 for 30 min, then stimulated with activin A (10 ng ml^–1^) or TGF-*β* (1 ng ml^–1^) for 1 h. The phosphorylation and expression levels of Smad2 were evaluated using a western blot. (**B**) KATOIII and MKN7 cells were cultured in 96-well plates and stimulated with the indicated doses of activin A or TGF-*β* for 72 h. Cellular proliferation was assayed using an MTT assay. Columns: mean of independent triplicate experiments. Bars: s.d.

Since whether activin A stimulation inhibits the cellular proliferation of GC cell lines as well as vascular endothelial cells remains unclear, we evaluated this effect. Activin A stimulation strongly upregulated the expression levels of p-smad2 in KATOIII cells, while it slightly upregulated them in MKN7 cells (Figure 6A). TGF-*β* potently upregulated the expression levels of p-smad2 in both cell lines. Regarding cellular proliferation, activin A inhibited cellular proliferation in KATOIII cells but did not inhibit proliferation in MKN7 cells, while TGF-*β* potently inhibited cellular proliferation when administered at a dose of 1–10 ng ml^–1^ (Figure 6B). These results indicate that activin A has a weak or no effect on the cellular proliferation of GC cells, compared with that of vascular endothelial cells.

### Overexpression of activin A inhibits tumour growth and angiogenesis *in vivo*

**Figure 7:**
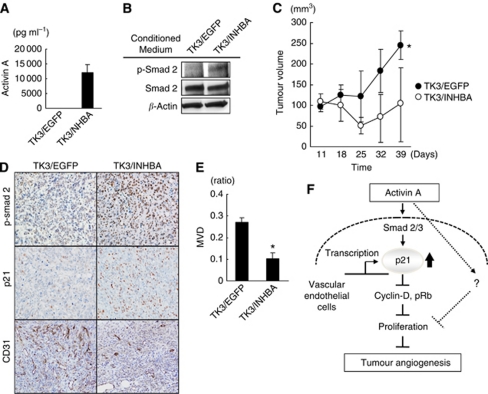
Overexpression of *INHBA* potently inhibited tumour growth and angiogenesis in GC *in vivo*. (**A**) TU-KATOIII GC cells were stably transfected with an *INHBA* (TK3/INHBA cells) or control vector (TK3/EGFP cells). Activin A secretion in the conditioned medium was analysed using an ELISA. (**B**) Activity of exogenous activin A on phosphorylation of smad2. Conditioned media from TK3/INHBA or /EGFP cells were exposed to HUVECs for 1 h and the phosphorylation of Smad2 in the HUVECs was assessed using a western blot analysis. *β*-Actin was used as an internal control. (**C**) Effect of overexpression of *INHBA* on tumour growth. TK3/EGFP and TK3/INHBA cells (1 × 10^7^ cells) were subcutaneously inoculated into mice and evaluated for tumour growth *in vivo*. The data indicate the mean±s.d. ^*^P<0.05. (**D** and **E**) CD31 staining for tumour specimens. Microvessel density (MVD) was evaluated using CD31-positive endothelial cells in tumour specimens using a computer-assisted image analysis. ^*^P<0.05. (**F**) Diagram of the proposed mechanism of activin A on vascular endothelial cells and in angiogenesis.

*In vitro* experiments showed that activin A inhibits the cellular growth of vascular endothelial cells. Next, we evaluated the effects of activin A overexpression in GC using an *in vivo* experiment. The *INHBA* or *EGFP* genes were stably introduced to TU-KATOIII, a low activin A-expressing GC cell line, to produce cell lines that were designated as TK3/INHBA and TK3/EGFP, respectively. The TK3/INHBA cells markedly secreted activin A (12.1 ng ml^–1^) protein into the culture medium, compared with a control cell line (Figure 7A). The conditioned medium from the TK3/INHBA cells, but not from the TK3/EGFP cells, induced the phosphorylation of Smad2 on HUVECs (Figure 7B). These results indicate that exogenous *INHBA* actually functions as activin A.

TK3/INHBA and TK3/EGFP cells were inoculated into mice, and tumour growth and angiogenesis were evaluated. The tumour volume of the TK3/INHBA cells on day 39 was significantly smaller (104.9±86.2 mm^3^) than that of the TK3/EGFP cells (245.1±34.7 mm^3^; Figure 7C). These results clearly indicated that the overexpression of activin A in GC significantly inhibited tumour growth. Next, we evaluated angiogenesis in these tumour xenografts using CD31 staining. The microvessel density was significantly reduced in the tumours of TK3/INHBA cells, compared with those of TK3/EGFP cells (Figure 7D and E). Meanwhile, the expressions of p-smad2 and p21 were clearly elevated in cancer cells in TK3/INHBA-inoculated tumours, compared with the expression levels in TK3/EGFP cells. These results indicate that the overexpression of activin A upregulated the expression levels of p-smad2 and p21 in cancer cells *in vivo* (Figure 7D). These results show that the overexpression of activin A suppresses angiogenesis *in vivo* as it does *in vitro*. In conclusion, we found that activin A is overexpressed in GC, inhibiting the cellular proliferation of vascular endothelial cells via direct p21 induction and suppressing tumour growth and angiogenesis *in vivo* (Figure 7F). Our results provide insight into activin A and angiogenesis in GC.

## Discussion

TGF-*β* is almost definitively considered to be an angiogenic factor (Wakefield and Roberts, 2002), but the role of activin A remains largely unclear. Our results showed that activin A and TGF-*β* have different effects on the proliferation of vascular endothelial cells *in vitro*. In line with this difference, previous knockout mice studies have demonstrated that embryos lacking any one of the TGF-*β* signalling components die during mid-gestation as a result of impaired vascular development, exhibiting hyper-dilated, leaky vessels, and highlighting the importance of TGF-*β* signalling in the vascular system (Goumans *et al*, 2009). Meanwhile, activin-*β* A-deficient mice did not develop any defects in angiogenesis (Matzuk *et al*, 1995). Although we did not directly compare the effect on angiogenesis between activin A and TGF-*β in vivo*, our findings support the inhibitory role of activin A on tumour angiogenesis, unlike TGF-*β*.

The relation between activin A expression and clinical outcome remains controversial. A high expression of *INHBA* was associated with a favourable prognostic outcome, exerting a tumour suppressor and anti-angiogenic role in neuroblastoma patients (Schramm *et al*, 2005). Meanwhile, its expression was correlated with tumour aggressiveness and a poor clinical outcome in patients with oesophageal carcinoma (Yoshinaga *et al*, 2004). This discrepancy may be explained by the dual role of TGF-*β* signalling as a tumour suppressor and pro-oncogenic factor. The TGF-*β* signalling pathway has a complicated role in cancer cells, mediating the ability of the cells to participate negatively or positively in growth inhibition, proliferation, replication, invasion, metastasis, apoptosis, immune surveillance, and angiogenesis (Jakowlew, 2006). For example, a defect of function in the TGF-*β* signalling component leads to carcinogenesis by acting as a definite tumour suppressor during the early phase, but it may exhibit an oncogenic function during later clinical stages (Jakowlew, 2006). This context dependency of TGF-*β* signalling and differences in the origins of the cancer tissue may lead to such discrepancies. We plan to clarify the clinical meaning of activin A expression in GC in a future study.

Our findings and other reported data indicate that activin A inhibits the cellular proliferation of vascular endothelial cells (Panopoulou *et al*, 2005; Schramm *et al*, 2005): however, the underlying mechanism in endothelial cells has not been fully elucidated. p21 is a member of the cip/kip family of cyclin kinase inhibitors, and initial reports have demonstrated that p21 functions as a G1 cyclin kinase inhibitor and a downstream molecule of p53 (el-Deiry *et al*, 1993). p21 possesses a variety of cellular functions, including the negative modulation of cell-cycle progression, cellular differentiation, and the regulation of p53-dependent anti-apoptosis (reviewed in Garner and Raj, 2008). We demonstrated that activin A directly regulates p21 expression at the transcriptional level, and the knockdown of p21 increased the cellular proliferation and mediated the resistance to activin A-mediated growth inhibition. Our findings have thus shed light on p21 as an activin A-mediated growth inhibitor in vascular endothelial cells.

In conclusion, our findings indicate that activin A inhibits vascular endothelial cell growth via the direct induction of p21 and highlight the suppressive role of activin A in tumour growth and angiogenesis in GC.
